# Effect of leptin C528T and leptin C73T polymorphisms and pregnancy on adipose tissue formation and carcass grade in Aberdeen Angus heifers and first-calf cows

**DOI:** 10.14202/vetworld.2022.1632-1640

**Published:** 2022-07-08

**Authors:** K. M. Dzhulamanov, S. V. Lebedev, Nikolay Gerasimov, V. I. Kolpakov

**Affiliations:** Federal Research Centre of Biological Systems and Agrotechnologies RAS, Orenburg, Russian Federation

**Keywords:** Aberdeen Angus, beef cattle, genotyping, leptin, polymorphism

## Abstract

**Background and Aim:**

The leptin (LEP) gene plays a role in the regulation of the activity required to obtain food, energy metabolism, and fat deposition and affects the body composition of animals. Lipogenesis is an ineffective process. A lot of energy from feed is expended on the synthesis of adipose tissue. This study aimed to determine the effect of LEP C528T and LEP C73T polymorphisms and pregnancy on adipose tissue formation and carcass grade in Aberdeen Angus heifers and first-calf cows.

**Materials and Methods:**

Heifers (n = 49) and first-calf cows (n = 30) were grouped according to their genotype for LEP C528T and LEP C73T polymorphisms at the age of 24 months. DNA samples were isolated from whole blood. Experimental animals were slaughtered at the age of 24 months, and a chemical analysis of samples of longissimus dorsi muscle and ground beef was performed.

**Results:**

The maximum internal fat weight, back-fat thickness, and fat content of the longissimus dorsi muscle and ground beef were determined in heifers heterozygous for both LEP C528T and LEP C73T polymorphisms. The ranking of genotypes in terms of adipose tissue formation did not change in first-calf cows compared to heifers carrying the same LEP gene variants. Pregnancy had a more significant (p < 0.05–0.001) effect on slaughter parameters and lipogenesis in animals of different genotypes than studied polymorphisms. Heterozygosity of the LEP gene was much more strongly expressed in the carcass grade of heifers. ”Prime” grades were assigned to 66.7% of carcasses heterozygous for LEP C528T and “Top Choice” to 58.8% of carcasses with LEP C73T nucleotide substitutions.

**Conclusion:**

LEP C528T and LEP C73T polymorphisms were shown to affect the extent of fat formation in Aberdeen Angus heifers and first-calf cows. Animals with heterozygous genotypes exhibited the maximum development of internal fat, back-fat, and intramuscular fat. Pregnancy had a more significant effect on slaughter parameters and adipose tissue formation than studied polymorphisms. First-calf cows had a significantly lower fat content in carcasses than heifers. These results can aid in the production of efficient mature herds of Aberdeen Angus cattle.

## Introduction

The Aberdeen Angus breed is the most popular beef breed in Russia as well as worldwide. Aberdeen Angus heifers have achieved popularity in Russia due to their high productivity and excellent meat quality. This breed competes with Herefords in the domestic market, which are also an important part of the country’s livestock. Russia occupies a large territory and, therefore, includes many different ecological and climatic zones. The Aberdeen Angus breed is primarily farmed in areas with a mild climate and good pastures, such as the southwestern and central parts of the country. The production of Aberdeen Angus cattle is maximized in these zones. Other breeds, such as domestic Kalmyk and Kazakh white-headed breeds, are best adapted to dry steppe areas and sharply continental climates with extremely cold winters and very hot summers. Aberdeen Angus cattle cannot compete with these breeds in these areas because of their decreased productivity and reproductive ability. Therefore, the Aberdeen Angus breed is widely used in crossbreeding programs to improve the productive traits of native breeds in these zones. Consumer demand for high-quality beef is becoming a major driving force in beef cattle breeding [1]. However, selection and breeding in domestic herds are hampered by the correctness of the lifetime assessment and the ability to select animals for the nutritional value of their meat because this can only be done after the slaughter of animals [2, 3]. At present, this problem is addressed by genotyping the livestock and selecting carriers of desirable genotypes, including genes associated with the qualitative characteristics of beef [4, 5]. The search for associations between substitutions in the DNA and productive traits is becoming important in the breeding of farm animals. Genotyping allows increased accuracy of assessment and selection, prediction of productivity, and culling of individuals unsuitable for breeding, thereby significantly reducing production costs [6–8]. However, the identification of candidate genes that are associated with the phenotypic variability of animals is a major issue [9]. The leptin (LEP) gene is a promising gene in cattle breeding. It is characterized by a high degree of polymorphism and is associated with important functions, such as fat and energy metabolism, feeding behavior, milk productivity, feed efficiency, and reproductive ability [10–12]. The LEP gene has been identified on bovine chromosome 4. The product of this gene is the hormone LEP, which is synthesized by adipocytes [13, 14]. Nucleotide substitutions in different regions of the gene affect the serum concentration of LEP [15, 16], which affects the phenotype, productive traits, and reproductive function of cattle [17–19]. According to Houseknecht *et al*. [20] and Baile *et al*. [21], the LEP gene plays a role in regulating food activity and energy metabolism and affects the body composition of animals.

The amount and rate of fat deposition and the peculiarities of lipid metabolism are genetically determined traits that affect the rate of weight gain, time to maturity, body conformation, and quality of meat and dairy products in cattle [22–24]. Lipogenesis is an ineffective process from an energy and economic point of view, and plenty of feed energy is expended on the synthesis of adipose tissue. Therefore, evaluating the relationship between the rate of fat deposition and genetic variability depending on the physiological status of animals will make it possible to create energy-efficient mature herds.

This study aimed to determine the effect of LEP C528T and LEP C73T polymorphisms and pregnancy on adipose tissue formation and carcass grade in Aberdeen Angus heifers and first-calf cows.

## Materials and Methods

### Ethical approval

All animal studies were performed in accordance with the instructions and recommendations of Russian Regulations, 1987 (Order No. 755 on 12.08.1977 the USSR Ministry of Health) and “The Guide for Care and Use of Laboratory Animals (National Academy Press Washington, D.C. 1996).” Every possible effort was made during the research to minimize animals’ distress and use fewer samples.

### Study period and location

The study was carried out from August to October 2021 at the feedlot and meat processing plant of the Zarechnoye Group of Companies (Ramonsky District, Voronezh Region, Russia).

### Sample collection and analysis

DNA samples were isolated from whole blood using a commercial PROBA-GS kit (DNA Technology, Russia), according to the manufacturer’s instructions. The quality of DNA samples was evaluated using horizontal electrophoresis “SE-2” (“Helicon,” Russia) in 1.5% agarose gel “Agarose, Biotechnology grade” (Helicon).

A quantitative polymerase chain reaction (PCR) was performed using Bio-Rad CFX 96 (Bio-Rad, Hercules, CA, USA). The following components were mixed in a 0.2 mL tube: 5 μL of ready-made PCR mixture qPCRmix-HS (Eurogen, Russia), 1 μL of 5 μmol forward primer, 1 μL of 5 μmol reverse primer, 1 μL of each of the two probes (5 μmol), 2 μL of DNA matrix, and up to 25 μL of deionized water. PCR was performed according to the following protocol: Initial heating at 37°C for 5 min, followed by denaturation at 94°C for 5 min, 40 cycles at 94°C for 15 s, and 1 cycle at 60°C for 1 min.

The following primers and probes were used for single-nucleotide polymorphism genotyping:

1. We used previously developed nucleotide sequences of primers and probes to identify LEP C528T at the LEP gene promoter [16]:
  - F: 5′-AGGTGCCCAGGGACTCA-3′
  - R: 5′-CAACAAAGGCCGTGTGACA-3′
  - Probe 1: FAM-CAAGCTCTAGAGCCTGTG T-BHQ1
  - Probe 2: HEX-AAGCTCTAGAGCCTAT GT-BHQ1.
2. The following primers and probes were designed by us and used to identify LEP C73T in exon 2 of the LEP gene [25]:
  - F: 5′-GGACCCCTGTWTCGATTCCT-3′
  - R: 5′-TGTCTTGATGAGGGTTTTGG-3′
  - Probe 1: FAM-CTGTGCCCATCCGCAAG GTCCA-BHQ1
  - Probe 2: HEX-CTGTGCCCATCTGCAAG GTCCA-BHQ1.

### Animals

Heifers (n = 49) and first-calf cows (n = 30) were grouped according to their genotype for LEP C528T and LEP C73T polymorphisms at the age of 24 months. The same conditions of feeding and rearing were maintained for all animals. The animals were reared according to the traditional technology for specialized beef cattle breeding as follows: In winter, they were kept loose in unheated rooms on straw underlay, whereas in summer, they were grazed on natural pastures. Experimental animals were slaughtered at the age of 24 months according to State Standard R 34120-2017 “Cattle for slaughter. Beef and veal in carcasses, half carcasses, and quarters.” [26] Carcasses were dressed and deboned according to State Standard R 31797-2012 “Meat. Cutting beef into cuts.” [27] The carcasses were graded according to the American standards [28]. The determination of marbling of meat products was assessed according to the development of intramuscular fat on the section of the longissimus dorsi muscle between the 12^th^ and 13^th^ ribs [29]. Samples of ground beef weighing 400 g were collected from left half-carcasses. Before deboning, 200 g samples of the longissimus dorsi muscle were collected from the same half-carcass at the level of the 9^th^–11^th^ ribs. The samples of longissimus dorsi muscle and ground beef were subjected to chemical analysis. Fat was extracted using a Soxhlet extraction unit as described by the Association of Analytical Communities [30], and the fat content was determined.

### Statistical analysis

The effects of genotype on the traits studied were analyzed using the least-squares method using the general linear model procedure of the Statistica 10.0 software (“Stat Soft Inc.,” USA).

Model used:

Y_ij_ = m + A_i_ + B_j_ + e_ij_

Where, Y_ij_ – represents the studied traits, μ – is the overall mean, A_i_ – is the fixed effect of the LEP genotype (1, 2, 3), B_j_ – is the fixed effect of the physiological status, and e_ij_ – is random error.

The effects of sire and dam on the performance of the offspring were not incorporated into the statistical model because we used a single sire for the artificial insemination of cows of the same age, from the same herd. The significance of inter-genotype differences was assessed using a posteriori Tukey’s criterion for unequal N. p ≤ 0.05 was considered statistically significant.

## Results

Animals heterozygous for the LEP C528T polymorphism had the maximum pre-slaughter live weight, that is, 558.0 kg, which exceeded that of cattle with the TT and CC homozygous genotypes by 2.9–5.4% (p > 0.05 for TT and p < 0.05 for CC) (Table-1). The distribution of heifers’ genotypes by carcass weight was the same as the ranking obtained according to the pre-slaughter live weight. Thus, animals with the CT genotype had the maximum carcass weight, which differed from that of homozygous animals by 2.5–3.0% (p > 0.05 for TT and p < 0.05 for CC).

**Table 1:** Adipose tissue development in Aberdeen Angus heifers depending on LEP С528T and LEP C73T polymorphism (M ± m).

Indicator	LEP C528T			LEP C73T		
	
	CC (13)	CT (24)	TT (12)	CC (17)	CT (17)	TT (15)
Live weight, kg	527.6 ± 6.45^a^	558.0 ± 7.87^a^	541.7 ± 5.67	545.0 ± 7.42	554.1 ± 8.55	539.9 ± 8.49
Carcass weight, kg	312.0 ± 5.38	321.8 ± 4.51	313.5 ± 4.19	316.7 ± 4.74	319.1 ± 5.54	316.4 ± 4.75
Internal fat weight, kg	14.11 ± 0.94^a^	16.59 ± 0.55^a^	15.37 ± 0.60	16.20 ± 0.52	15.58 ± 0.61	15.04 ± 1.01
Internal fat yield, %	2.69 ± 0.19	2.99 ± 0.11	2.84 ± 0.11	2.98 ± 0.10	2.83 ± 0.13	2.80 ± 0.19
Back-fat thickness, mm						
In the middle of the last rib	4.11 ± 0.21^a^	5.18 ± 0.21^a^	4.32 ± 0.27	4.47 ± 0.26	4.84 ± 0.24	4.75 ± 0.29
On the 3^rd^ lumbar vertebra	10.42 ± 0.53	11.65 ± 0.44	11.35 ± 0.48	10.96 ± 0.37	11.92 ± 0.53	10.93 ± 0.58
At the tail root	19.22 ± 0.64	19.30 ± 0.52	19.32 ± 0.54	18.75 ± 0.56	19.98 ± 0.56	19.09 ± 0.56
Fat content, %						
Longissimus dorsi muscle	3.58 ± 0.100	3.73 ± 0.73	3.64 ± 0.095	3.59 ± 0.067	3.84 ± 0.091	3.58 ± 0.090
Ground beef	23.75 ± 0.840	25.44 ± 0.480	23.97 ± 0.506	24.22 ± 0.502	24.90 ± 0.541	24.80 ± 0.843

a Values in a row with the same indices differ with significance P< 0.05. LEP=Leptin, CC=genotype CC, CT=genotype CT, TT=genotype TT

Significant differences were found in adipose tissue development. Heifers with the LEP C528T^CT^ genotype had significantly higher weight and more internal fat than the homozygotes by 1.22–2.48 kg and 0.15–0.18%, respectively (p > 0.05 and p < 0.05) compared with TT and CC genotypes.

The location of subcutaneous adipose tissue affected the intergroup variability in back-fat thickness. The back-fat thickness did not differ significantly at the tail root in heifers. Young animals with the LEP C528T^CC^ genotype had 1.07 cm (p < 0.05) less back-fat in the middle of the last rib than those with the heterozygous genotype. Young animals with the LEP C528T^CT^ genotype had 0.30–1.23 cm greater thickness of the subcutaneous fat on the lower back than homozygotes.

Chemical analysis of slaughter products indicated that LEP C528T^CT^ individuals produced more fat; that is, they had higher fat content in the longissimus dorsi muscle and ground beef by 0.09–0.15% and 1.47–1.69%, respectively, than in peers homozygous for the LEP C528T polymorphism.

Analysis of the impact of LEP C73T polymorphism showed that it had a significantly smaller contribution to the variability in slaughter parameters and adipose tissue development in Aberdeen Angus heifers. Animals with the homozygous genotype LEP C73T^CC^ had 0.62–1.16 kg more internal fat. The maximum visceral fat yield was also found in heifers from this group. However, heterozygous individuals had higher subcutaneous adipose tissue formation. They exceeded their peers in back-fat thickness at the last rib point by 0.09–0.37 mm, at the lower back by 0.96–0.99 mm, and at the tail root by 0.89–1.23 mm. Further, fat synthesis increased by 0.25–0.26% in the longissimus dorsi muscle and by 0.10–0.68% in ground beef in Aberdeen Angus heifers with the LEP C73T^CT^ genotype.

Pregnancy had a more significant effect on slaughter parameters and lipogenesis in animals of different genotypes than studied polymorphisms (Table-2). The production of offspring led to a 21.7–46.9 kg (p < 0.01–0.001) decrease in the carcass weight of first-calf cows compared to heifers carrying the respective genotypes with the LEP C528T polymorphism and a 5.8–55.7 kg (p > 0.05 and p < 0.001) decrease in the carcass weight of those with the LEP C73T polymorphism.

**Table 2:** Adipose tissue development in Aberdeen Angus first-calf cows depending on LEP С528T and LEP C73T polymorphism (M ± m).

Indicator	LEP C528T			LEP C73T		
	
	CC (12)	CT (15)	TT (3)	CC (3)	CT (16)	TT (11)
Live weight, kg	524.3 ± 19.62	548.8 ± 24.71	549.5 ± 54.28	585.2 ± 73.94	547.7 ± 22.06	514.0 ± 18.35
Carcass weight, kg	265.1 ± 10.76	278.6 ± 14.14	291.8 ± 35.81	310.9 ± 43.67	277.2 ± 12.67	260.7 ± 10.03
Internal fat weight, kg	9.29 ± 0.50	10.31 ± 0.48	9.57 ± 0.61	10.10 ± 70.75	10.17 ± 0.42	9.25 ± 0.59
Internal fat yield, %	1.76 ± 0.06	1.89 ± 0.06	1.76 ± 0.10	1.75 ± 0.10	1.87 ± 0.05	1.79 ± 0.08
Back-fat thickness, mm						
In the middle of the last rib	3.22 ± 0.13^ab^	4.47 ± 0.10^a^	4.40 ± 0.23^b^	4.57 ± 0.28	4.10 ± 0.16	3.60 ± 0.25
On the 3^rd^ lumbar vertebra1	8.58 ± 0.26	9.62 ± 0.46	9.43 ± 0.50	10.50 ± 1.25	9.18 ± 0.37	8.84 ± 0.37
At the tail root	15.48 ± 0.80	15.92 ± 0.53	15.46 ± 1.09	16.60 ± 1.72	15.85 ± 0.48	15.24 ± 0.82
Fat content, %						
Longissimus dorsi muscle	2.79 ± 0.073^a^	3.15 ± 0.085^a^	3.05 ± 0.104	3.13 ± 0.148	3.00 ± 0.058	2.95 ± 0.141
Ground beef	16.49 ± 0.613	18.27 ± 0.426	17.77 ± 0.899	18.79 ± 1.298	17.44 ± 0.597	17.26 ± 0.339

a Values in a row with the same indices differ with significance P< 0.05,

b -P< 0.05. LEP=Leptin, CC=genotype CC,

CT=genotype CT, TT=genotype TT

The visceral fat weight and its yield increased significantly by 4.82–6.28 kg (p < 0.001) and 0.93–1.10% (p < 0.001), respectively, in heifers with the LEP C528T polymorphism, and by 5.41–6.10 kg (p < 0.01–0.001) and 0.96–1.23% (p < 0.01–0.001), respectively, in those with the LEP C73T polymorphism. Heifers of all genotypes showed significantly high subcutaneous adipose tissue development, regardless of the measurement point (p < 0.05–0.001).

The chemical composition of the longissimus dorsi muscle and ground beef showed significant differences in fat content between samples from heifers and first-calf cows with certain genotypes. The longissimus dorsi muscle in heifers with the LEP C528T and LEP C73T polymorphisms of the LEP gene contained 0.58–0.79% (p < 0.01–0.001) and 0.46–0.63% (p > 0.05 and p < 0.001) more fat, respectively, than first-calf cows; these differences were 6.20–7.26% (p < 0.001) and 5.43–7.54% (p < 0.01–0.001), respectively, in ground beef, in favor of heifers.

The effect of LEP C528T polymorphism on the rate of fat deposition in first-calf cows had a similar ranking of genotype distribution as that established for heifers. Heterozygous LEP C528T^CT^ individuals had superior slaughter parameters and adipose tissue development compared to heifers. The most significant differences were found in back-fat thickness at the last rib point, which was greater by 1.25 mm (p < 0.001), and in the fat content of the longissimus dorsi muscle, which was higher by 0.36% (p < 0.05). However, LEP C73T polymorphism had less effect on the variability of the studied parameters.

The amount of fat deposition in heifers with the LEP C528T^CT^ genotype was confirmed by the morphological composition of particular cuts (Table-3). These animals surpassed their peers both in absolute weight and in the yield of intermuscular adipose tissue in cuts. The lowest accumulation of fat was observed in the cuts of homozygous LEP C528T^CC^ individuals. The differences between extreme variants in weight and in the proportion of neck fat were 0.6 kg (p < 0.05) and 2.4% (p < 0.01), respectively. The corresponding indicators were 1.1 kg (p < 0.01) and 2.0% (p < 0.01) in spinal cuts. A non-significant increase in heifers heterozygous for the LEP C528T polymorphism was recorded in other parts of the carcass. Carriers of the LEP C528T^TT^ genotype occupied an intermediate position in fat accumulation in some cuts. The highest total fat content in the carcass was also observed in heterozygous LEP C528T^CT^ heifers, which outperformed carriers of the LEP C528T^CC^ genotype by 2.8 kg (p < 0.05).

**Table 3:** Fat content in particular anatomic cuts in heifers depending on LEP С528T and LEP C73T polymorphism (M ± m).

Fat content in cuts	LEP C528T			LEP C73T		
	
	CC	CT	TT	CC	CT	TT
Neck, kg	1.7 ± 0.10^a^	2.3 ± 0.10^a^	1.9 ± 0.13	2.1 ± 0.11	2.1 ± 0.13	2.1 ± 0.15
%	9.3 ± 0.45^a^	11.7 ± 0.37^a^	10.1 ± 0.55	10.7 ± 0.43	10.7 ± 0.49	10.7 ± 0.64
Humeral-scapular, kg	2.0 ± 0.11	2.4 ± 0.10	2.1 ± 0.17	2.1 ± 0.14	2.3 ± 0.11	2.2 ± 0.14
%	6.5 ± 0.28	7.6 ± 0.25	7.0 ± 0.49	6.9 ± 0.38	7.4 ± 0.28	7.2 ± 0.37
Spinal, kg	1.5 ± 0.13^a^	2.6 ± 0.19^a^	1.9 ± 0.17	2.3 ± 0.27	2.3 ± 0.17	1.8 ± 0.16
%	3.6 ± 0.25^a^	5.6 ± 0.35^a^	4.4 ± 0.34	5.1 ± 0.48	5.2 ± 0.34	4.0 ± 0.32
Lumbar, kg	1.2 ± 0.07	1.4 ± 0.08	1.2 ± 0.07	1.3 ± 0.08	1.3 ± 0.10	1.3 ± 0.07
%	11.1 ± 0.47	12.6 ± 0.58	11.6 ± 0.52	11.9 ± 0.54	12.2 ± 0.71	12.2 ± 0.48
Hip, kg	3.2 ± 0.19	3.8 ± 0.23	3.6 ± 0.24	3.5 ± 0.24	3.6 ± 0.25	3.6 ± 0.23
%	6.3 ± 0.29	7.1 ± 0.33	7.4 ± 0.39	6.8 ± 0.36	6.9 ± 0.39	6.8 ± 0.35
Total fat in carcass, kg	9.7 ± 0.54^a^	12.5 ± 0.57^a^	10.8 ± 0.69	11.3 ± 0.70	11.7 ± 0.65	11.0 ± 0.69
%	6.3 ± 0.26^a^	7.8 ± 0.25^a^	7.0 ± 0.35	7.2 ± 0.34	7.4 ± 0.29	7.0 ± 0.34

a Values in a row with the same indices differ with significance P< 0.05. LEP=Leptin, CC=genotype CC, CT=genotype CT, TT=genotype TT

LEP C73T polymorphism did not significantly affect the accumulation of adipose tissue in particular carcass cuts in heifers. A small increase in fat content was observed in heterozygous LEP C73T^CT^ individuals.

Adipose tissue development in heifers was higher than in first-calf cows due to the consumption of energy, feed nutrients for fetal growth, subsequent milk production, and stress after weaning calves (Table-4).

**Table 4:** Fat content in particular anatomic cuts in first-calf cows depending on LEP С528T and LEP C73T polymorphism (M ± m).

Fat content in cuts	LEP C528T			LEP C73T		
	
	CC	CT	TT	CC	CT	TT
Neck, kg	1.3 ± 0.10	1.4 ± 0.14	1.4 ± 0.21	1.7 ± 0.39	1.4 ± 0.12	1.2 ± 0.09
%	7.9 ± 0.37	8.1 ± 0.43	8.0 ± 0.23	9.0 ± 0.97	8.2 ± 0.35	7.5 ± 0.39
Humeral-scapular, kg	1.3 ± 0.11	1.5 ± 0.16	1.5 ± 0.26	1.8 ± 0.42	1.4 ± 0.15	1.2 ± 0.09
%	5.0 ± 0.25	5.2 ± 0.38	5.2 ± 0.30	5.9 ± 0.67	5.1 ± 0.33	4.9 ± 0.27
Spinal, kg	1.0 ± 0.11	1.2 ± 0.14	1.2 ± 0.27	1.4 ± 0.35	1.2 ± 0.13	1.0 ± 0.09
%	2.7 ± 0.19	3.1 ± 0.23	3.0 ± 0.32	3.3 ± 0.41	3.0 ± 0.23	2.8 ± 0.19
Lumbar, kg	0.8 ± 0.07	0.9 ± 0.08	0.9 ± 0.18	1.1 ± 0.24	0.9 ± 0.06	0.8 ± 0.07
%	9.3 ± 0.46	9.7 ± 0.35	9.5 ± 0.75	10.2 ± 0.97	9.8 ± 0.29	8.8 ± 0.46
Hip, kg	2.1 ± 0.15	2.4 ± 0.23	2.4 ± 0.48	2.9 ± 0.70	2.3 ± 0.20	2.0 ± 0.11
%	4.8 ± 0.16	5.1 ± 0.28	5.0 ± 0.42	5.6 ± 0.68	5.1 ± 0.24	4.7 ± 0.14
Total fat in carcass, kg	6.6 ± 0.51	7.4 ± 0.72	7.5 ± 1.41	9.0 ± 2.09	7.3 ± 0.64	6.2 ± 0.39
%	5.0 ± 0.19	5.3 ± 0.26	5.2 ± 0.36	5.7 ± 0.64	5.2 ± 0.24	4.9 ± 0.13

LEP=Leptin, CC=genotype CC, CT=genotype CT, TT=genotype TT

These biological processes led to significant differences in fat synthesis between heifers and first-calf cows in some cuts. Around 3.3–5.1 kg (p < 0.001) more adipose tissue was collected from the carcasses of heifers depending on the same genotype of LEP C528T polymorphism. The fat proportion was 1.3–2.5% (p < 0.001) lower in carcasses from first-calf cows. The corresponding parameters for the LEP C73T polymorphism were 2.3–4.8 kg (p > 0.05) and 1.5–2.2% (p > 0.05). There was no significant effect of LEP gene polymorphism on adipose tissue formation in carcasses from first-calf cows.

**Figure-1:**
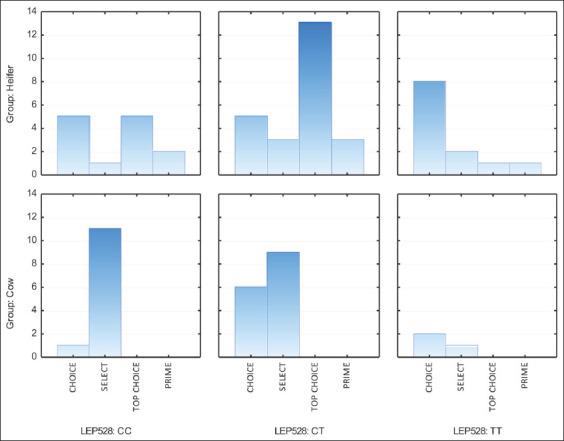
Effect of leptin C528T polymorphism on carcass grade in Aberdeen Angus first-calf cows and heifers.

**Figure-2:**
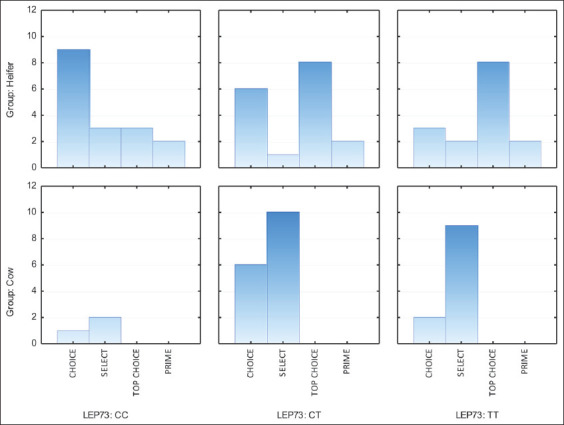
Effect of leptin C73T polymorphism on carcass grade in Aberdeen Angus first-calf cows and heifers.

The evaluation of the grade of carcasses indicated that the gradation of marbling was significantly affected by both the physiological condition and genotype of Aberdeen Angus cows and heifers (Figures-1 and 2).

Beef from first-calf cows did not achieve the highest grade categories, that is, “Top Choice” and “Prime.” In carcasses of Aberdeen Angus heifers, 12.24% (n = 6) met the “Prime” standard and 38.78% (n = 19) were “Top Choice.” Thus, heifers showed good marbling and high carcass yield.

The distribution of carcasses into categories according to genotype was uneven. A nucleotide substitution in the promoter region (LEP C528T) producing a heterozygous CT genotype was associated with better carcass quality in first-calf cows.

There was a positive effect of the C allele in the promoter region (LEP C528T) on beef marbling in Aberdeen Angus heifers. LEP C528T^TT^ carriers had the lowest grade; only 16.7% of individuals were assigned to the highest categories of “Top Choice” (8.33%) or “Prime” (8.33%). Only a third of carcasses (33.3%) from heifers with the heterozygous CT genotype were assigned to the lower classes “Select” (12.5%) and “Choice” (20.8%), and more than half (54.2%) belonged to the “Top Choice” category. The maximum proportion (16.7%) of “Prime” carcasses was obtained from carriers of the LEP C528T^CC^ genotype. The excess of “Prime” carcasses relative to other gene variants was 4.2–8.3%.

LEP C73T genotyping showed a similar distribution of carcass grade. However, the T allele in the region of exon 2 produced higher beef quality. Heifers with the LEP C73T^TT^ genotype, with a nucleotide substitution in the region of the second exon (LEP C73T), showed the best meat marbling. The highest carcass grade “Prime” was found 1.5% more often among the carriers of this variant of the LEP gene than in their peers with the CT and CC genotypes. A significant increase of 6.2–35.7% was observed in the proportion of “Top Choice” carcasses. Overall, 66.7% of the carcasses obtained from heifers with the homozygous LEP C73T^TT^ genotype were of the highest grades, “Top Choice” or “Prime.” Many (58.8%) of the most valuable carcasses had the heterozygous LEP C73T^CT^ genotype.

## Discussion

Variability in the LEP gene was first reported in humans and mice [31, 32]. Subsequently, nucleotide substitutions in the LEP gene were detected in many farm animals, making it possible to establish an association between its polymorphism and the development of a wide range of economically important traits [10, 33]. We evaluated the association of the single-nucleotide polymorphisms LEP C73T and LEP C528T of the LEP gene with the development of adipose tissue in Aberdeen Angus first-calf cows and heifers. The polymorphism LEP C73T (Arg25Cys, R25C or 305C > T) occurs in exon 2 at codon 25 of the gene, leading to the functional substitution of arginine for cysteine. Consequently, the N-terminal signal peptide is lost, inhibiting LEP secretion from adipose tissue [34]. This mutation is associated with fat content in the carcasses of beef cattle. In particular, cysteine synthesis is associated with high-fat content in carcasses and arginine with leaner carcasses [25]. According to several studies, an understanding of LEP C73T polymorphisms may be valuable in the selection and breeding of beef cattle. [35, 36]. Kononoff *et al*. [37] found a significant (p = 0.011) effect of nucleotide substitution in the second exon (R25C) on the formation of back-fat at the 12^th^ rib point and intramuscular fat. The homozygous TT genotype showed significant (p = 0.003) increases in dry matter intake, (p = 0.016) accumulation of raw fat, and meat yield (p = 0.020). In this study, increased back-fat thickness at different measurement points as well as higher fat content in the longissimus dorsi muscle and ground beef were observed in heifers with the heterozygous LEP C73T^CT^ genotype. However, the superiority of heterozygous individuals was statistically non-significant. The LEP C73T^TT^ genotype in heifers was associated with minimal internal fat content. The increased level of fat deposition in first-calf cows with the LEP C73T^CC^ genotype can be disregarded due to the small size of the sample. The ranking of LEP C73T^CT^ and LEP C73T^TT^ genotypes in terms of internal fat content, back-fat thickness, and fat content in the longissimus dorsi muscle and ground beef did not change in first-calf cows compared to heifers carrying the same LEP gene variants.

Changes in the nucleotide sequence in the LEP C528T region are much less important in marker-assisted selection than the LEP C73T polymorphism in the selection and breeding of cattle. This single-nucleotide polymorphism occurs in the 5′-untranslated region of the LEP gene promoter [38]. Nkrumah *et al*. [16] found that a mutation in the LEP C528T promoter region (UASMS2) of the LEP gene is associated with 31–39% (p < 0.001) increase in the synthesis of subcutaneous adipose tissue and 9–13% (p = 0.01) increase in meat marbling. Individuals with the TT genotype showed increased food consumption (p < 0.001), weight gain (p < 0.05), and pre-slaughter live weight (p < 0.10). In contrast, we found that heterozygous heifers exhibited the highest back-fat development, internal fat weight, and fat content in the longissimus dorsi muscle and ground beef, whereas homozygous TT genotype carriers exhibited intermediate expression of these traits. The least amount of adipose tissue development was observed in LEP C528T^CC^ carriers. The results of the effect of LEP C528T polymorphism in heifers were also confirmed in first-calf cows. The significant superiority (p < 0.05) of heterozygous genotype carriers was established by the back-fat thickness in the last rib point and the fat content of the longissimus dorsi muscle. The data obtained were in good agreement with the results of the study by Wang *et al*. [39], who found a significant effect of the UASMS2 (LEP C528T) polymorphism of the LEP gene on meat marbling and back-fat thickness.

Our data indicate the effect of variability in the LEP gene on the variability of live weight and slaughter traits in Aberdeen Angus first-calf cows and heifers. Similar studies were carried out by Lusk [40] when feeding crossbred bulls and heifers. He studied the association of UASMS2 (LEP C528T in our study) and R25C (LEP C73T in our study) polymorphisms with weight gain and age-related changes in back-fat thickness. There was a highly significant (p < 0.001) combined effect of R25C and UASMS2 on the variability of back-fat thickness. Thus, the R25C-CC/UASMS2-TT haplotype was associated with the lowest development of subcutaneous adipose tissue at the beginning of the experiment. However, later its carriers showed the maximum growth rate of adipose tissue. Animals with the R25C-CC/UASMS2-CC combination showed minimal age-related variability in back-fat thickness.

In another experiment, Kononoff *et al*. [41] evaluated the effect of Arg25Cys polymorphism on carcass grade in steers and heifers. There was a positive association of the TT genotype with the category of carcasses according to the Canadian classification, which the authors explained by the peculiarities of fat formation in carriers of different alleles. Genotyping revealed that individuals heterozygous for the polymorphisms studied produced the most valuable carcasses. Thus, 40% and 37.5% of carcasses from first-calf cows heterozygous for the LEP C528T and LEP C73T polymorphisms, respectively, were assigned to the “Choice” category. Heterozygosity of the LEP gene was much better expressed in the formation of carcass grade in heifers. “Prime” and “Top Choice” grades were assigned to 66.7% and 58.8% of carcasses with the LEP C528T and LEP C73T polymorphisms, respectively.

Nkrumah *et al*. [19] found a significant (p < 0.001) increase of 39–48% in the serum concentration of LEP in carriers of the UASMS2-TT genotype compared with those with the CT and CC genotypes. Garcia *et al*. [42] found a significant positive correlation between serum LEP and body fat in beef heifers.

In our study, the physiological state of the animals had a significantly greater effect on the intensity of fat metabolism than studied polymorphisms. Pregnancy had a negative effect on fat accumulation in the carcasses of first-calf cows compared to heifers of the same age due to the high-energy expenditure of bearing a fetus for carrying out vital functions [43].

## Conclusion

LEP C528T and LEP C73T polymorphisms affected the extent of fat formation in Aberdeen Angus heifers and first-calf cows. Heterozygous genotypes exhibited the maximum development of internal fat, back-fat, and intramuscular fat. Pregnancy had a more significant effect on slaughter parameters and adipose tissue formation than studied polymorphisms. First-calf cows had a significantly lower fat content in carcasses than heifers.

## Authors’ Contributions

KMD, SVL, NG, and VIK: Performed the experiments. KMD and NG: Drafted and edited the manuscript. SVL and VIK: Equally designed and conducted the study. NG and VIK: Studied scientific literature about the topic and drafted the manuscript. All authors have read and approved the final manuscript.
